# Uptake of *Plasmodium chabaudi* hemozoin drives Kupffer cell death and fuels superinfections

**DOI:** 10.1038/s41598-022-23858-7

**Published:** 2022-11-17

**Authors:** Isabella C. Hirako, Maísa Mota Antunes, Rafael Machado Rezende, Natália Satchiko Hojo-Souza, Maria Marta Figueiredo, Thomaz Dias, Helder Nakaya, Gustavo Batista Menezes, Ricardo Tostes Gazzinelli

**Affiliations:** 1 Instituto René Rachou, Fundação Oswaldo Cruz, Belo Horizonte, MG Brazil; 2 Division of Infectious Diseases and Immunology, Department of Medicine, University of Massachusetts Medical School, 364 Plantation Street, Lazare Research Building, 3rd Floor, Worcester, MA USA; 3 Ann Romney Center for Neurologic Diseases, Brigham and Women’s Hospital, Harvard Medical School, Boston, MA USA; 4 Center for Gastrointestinal Biology, Departamento de Morfologia, Instituto de Ciências Biológicas, Universidade Federal de Minas Gerais, Belo Horizonte, MG Brazil; 5 Departamento de Bioquímica E Imunologia, Instituto de Ciências Biológicas, Universidade Federal de Minas Gerais, Belo Horizonte, MG Brazil; 6 Universidade Estadual de Minas Gerais, Divinópolis, MG Brazil; 7 Escola de Ciências Farmacêuticas – Universidade de São Paulo, São Paulo, SP Brazil

**Keywords:** Immunology, Monocytes and macrophages

## Abstract

Kupffer cells (KCs) are self-maintained tissue-resident macrophages that line liver sinusoids and play an important role on host defense. It has been demonstrated that upon infection or intense liver inflammation, KCs might be severely depleted and replaced by immature monocytic cells; however, the mechanisms of cell death and the alterations on liver immunity against infections deserves further investigation. We explored the impact of acute *Plasmodium* infection on KC biology and on the hepatic immune response against secondary infections. Similar to patients, infection with *Plasmodium chabaudi* induced acute liver damage as determined by serum alanine aminotransferase (ALT) and aspartate aminotransferase (AST) elevation. This was associated with accumulation of hemozoin, increased of proinflammatory response and impaired bacterial and viral clearance, which led to pathogen spread to other organs. In line with this, mice infected with *Plasmodium* had enhanced mortality during secondary infections, which was associated with increased production of mitochondrial superoxide, lipid peroxidation and increased free iron within KCs—hallmarks of cell death by ferroptosis. Therefore, we revealed that accumulation of iron with KCs, triggered by uptake of circulating hemozoin, is a novel mechanism of macrophage depletion and liver inflammation during malaria, providing novel insights on host susceptibility to secondary infections. Malaria can cause severe liver damage, along with depletion of liver macrophages, which can predispose individuals to secondary infections and enhance the chances of death.

## Introduction

Malaria is one of the most deleterious infectious diseases in the world. According to the World Health Organization (WHO), there were an estimated 229 million malaria cases in 2019 in 87 endemic countries^1^, contributing to significant social and economic instability globally. Importantly, *Plasmodium* infections are co-endemic with diseases caused by other agents of acute febrile illnesses, such as bacteria and virus, and it is increasingly clear that superinfections with different microbial pathogens is a common occurrence that can enhance morbidity and mortality^2^. Studies demonstrate that infection with *Non-typhoidal Salmonella* (NTS) or pathogenic strains of *Escherichia coli* (*E. coli*) are the most frequent causes of community-acquired bacteremia identified in many patients in sub-Saharan Africa^3–6^. These co-infections have been associated with high malaria mortality among children^7–12^. Besides, the mortality of malaria patients co-infected with Gram negative bacteria is up to eight times higher than in individuals with malaria monoinfection^13^. In line with this, co-infection of mice with *Plasmodium chabaudi* (*P. chabaudi*), *Citrobacter rodentium* and *Salmonella typhimurium* drive an excessive host inflammatory response and increased lethality^14,15^. Additionally, it has been demonstrated that infection with murine gammaherpesvirus 68 (MHV68) can suppress the anti-malarial humoral response to a secondary malaria infection^16^, and co-infection with hepatitis B virus (HBV) or dengue—infections which are prevalent in most tropical countries—increase morbidity in malaria patients^17^. However, the mechanisms through which concomitant malaria and bacteria or virus co-infection drive excessive mortality remain unclear.

The liver plays a crucial role in immune surveillance throughout life, removing protein complexes, small particles, apoptotic cells from blood and microorganisms^18–20^. This organ is equipped with a large population of immune cells, including granulocytes, natural killer cells, dendritic cells, monocytes and liver macrophages (Kupffer cells; KCs). Due to their abundance and physiologically localization within the sinusoids, KCs represent the first barrier of defense against pathogens to enter the liver via the portal circulation^21^. The specialized function of KCs can be activated by pathogen associated molecular patterns (PAMPs) such as lipopolysaccharide (LPS), glycolipids, flagellin, lipoproteins, viral RNA and DNA as well as endogenous ligands^22^. When KCs phagocyte parasites, a variety of pro-inflammatory cytokine are released, initiating the acute-phase response and inflammation^23^. In addition, KCs express class II major histocompatibility complex molecules (MHCII), which is crucial for processing and presenting antigens, which are involved with the liver inflammation under different circumstances^24^.

In fact, upon *Plasmodium* infection, it is common to observe accumulation of hemozoin within hepatic cells—an insoluble crystal that results from hemoglobin digestion by the erythrocytic stages of *Plasmodium* parasites^25^. This is also associated with hepatic injury, inflammation and failure, which could contribute to evolution to severe forms of malaria^26–28^. Clinical manifestations such as hepatic dysfunction, jaundice and high serum levels of alanine transaminase (ALT) and aspartate transaminase (AST), are frequently observed, as already described^29,30^. Based on data showing that patients with *P. falciparum* malaria and mice experimentally infected with *Plasmodium*^28^ displayed histological signs of hyperplastic KCs containing hemozoin (malarial pigment), centrizonal hepatocellular necrosis with focal inflammation and degeneration^31^, here we investigated the impacts of murine *P. chabaudi* infection on KCs biology and function, and how such alterations on KCs profile can impact on the severity of co-infections in bacterial and viral models. Our results suggest a role of hemozoin inducing necrosis followed by KC ferroptosis, recruitment of leukocytes including a population of inflammatory monocytes, which were, however, inefficient in controlling superinfections with either bacteria or virus.

## Results

### *Plasmodium* infection triggers liver damage, inflammation and failure

**Figure 1:**
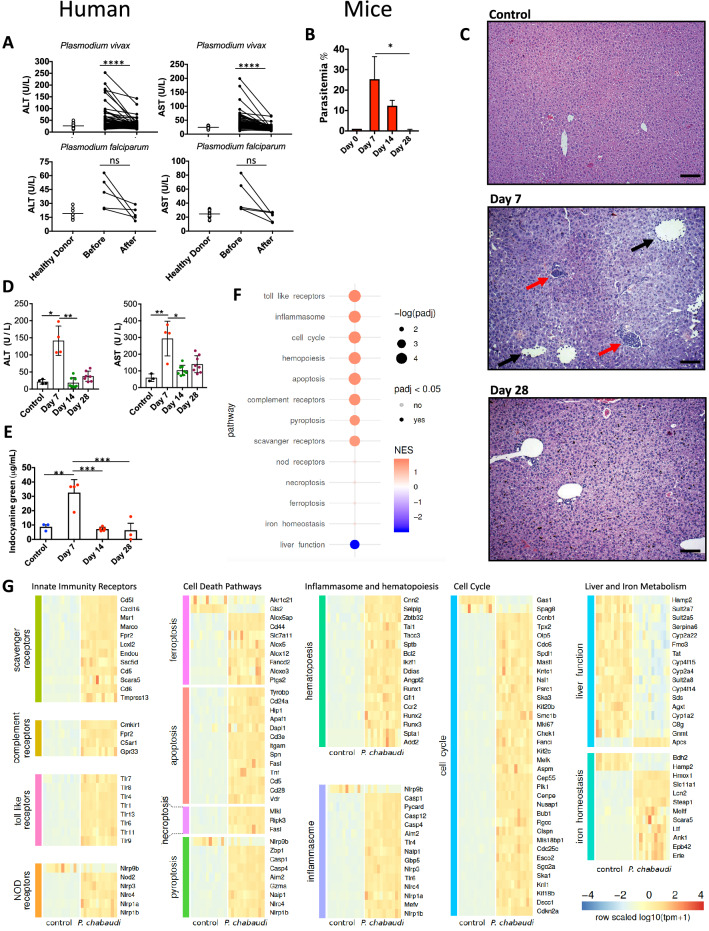
Liver injury during *Plasmodium* infection. (**A**) Human serum ALT and AST levels during acute phase and after treatment (control n = 16, *P. vivax* n = 54, *P. falciparum* n = 5). (**B**) Parasitemia (n = 6), (**C**) Liver histopathology (HE staining, Bar = 64 µm) and (**D**) Serum ALT and AST of control (n = 4) and infected mice at days 7 (n = 4), 14 (n = 8) and 28 (n = 8) post-infection. (**E**) Liver dysfunction during different times of infection (n = 3–5). (**F**) Genetic analysis of different pathways related to liver inflammation. (**G**) Heatmap of differential expression of genes involved in inflammation. Color scheme represents the global *Z score*. The data shown are representative of 3 independent experiments. Results expressed as mean ± SD. Statistical significance comparing infected and non-infected mice (Kruskal–Wallis test, *p < 0.05, **p < 0.01, ***p < 0.001, ****p < 0.0001).

*Plasmodium* infection in humans initiates with sporozoite invasion within hepatocytes, followed by a dramatic parasite multiplication in the liver. Each liver stage forms up to 90,000 exoerythrocytic merozoites^31^. Regardless, infection and parasite replication within hepatocytes is silent and at this stage infection is asymptomatic. It is the exponential growth of merozoites in the red blood cells that associates with liver damage and signs of disease. The liver alterations seem to be a consequence of hemozoin accumulation and local inflammation. However, the mechanistic details of this process and how such disturbances on hepatic immune response could explain enhanced susceptibility to superinfections during malaria is largely unknown. To address these questions, we first collected blood samples from symptomatic malaria patients infected with either *P. vivax* or *P. falciparum* and also after the end of pharmacological treatment and disease remission. As compared to healthy donors, infected patients with either *Plasmodium* species showed signs of liver damage, as indicated by serum levels of both ALT and AST (Fig. 1A). Patients diagnosed with either *P. vivax* or *P. falciparum* infection that received pharmacological intervention displayed remission of serum elevations of ALT and AST, as previously described, suggesting regression of liver injury^57^. Despite no statistically significance, we also observed an increase of liver enzymes in serum of the *P. falciparum* infected patients, and both AST and ALT had a 50–90% reduction after treatment (Fig. 1A). Together, these data suggest that malaria patients had higher chances to evolve with liver injury, which is recovered following parasite elimination.

To model human *Plasmodium*-induced hepatic injury in mice and further understand the putative impacts on immune function, we infected mice with *P. chabaudi,* a rodent variant whose pathology resembles human features of malaria, including anemia, hypoglycemia, weight loss, and hypothermia. Following 7 days of infection, mice presented a significant increase in parasitemia (presence of parasites inside red blood cells; Fig. 1B), which persisted for approximately 28 days, when parasitemia was completely cleared. In line with our data from malaria patients, infected mice also had histopathological alterations in liver samples at the peak of parasitemia (7 days after infection; Fig. 1C), with a massive area of hydropic degeneration displaying widespread leukocytic infiltration within sinusoids (Fig. 1C; black arrows) and leucocyte islets (yellow arrows). Accordingly, we also detected significant elevation on serum ALT and AST levels, connecting *Plasmodium* replication with hepatocyte damage (Fig. 1D). To dissect if such grade of liver damage could impact on liver metabolic function, we developed an experimental strategy where mice were given intravenously a known amount of indocyanine green (ICG), and its hepatic clearance capacity was measured real time using spectrophotometry. As shown in Fig. 1E, non-infected mice are fully able to remove ICG out of circulation since a very small amount was detected in their blood following 20 min of administration. In sharp contrast, mice in the peak of parasitemia and liver damage presented sustained higher levels of ICG in the blood, confirming a precarious clearance function by the liver at this timepoint. After parasite clearance (14^th^ to 28^th^ days after infection), hepatic depurative function returned to baseline values and full function was restored. Taken together, these data connect *Plasmodium* infection with higher chances of liver injury and failure, allowing further investigation using this mouse model.

There is a growing body of evidence showing that hepatic immune system can rapidly react to necrosis product and parenchymal damage^24^. In fact, liver harbors one of the most complex immune environment in the body, and virtually all leukocyte subpopulations can be found within the hepatic environment under physiological conditions^35,36,38^. Based on this premise, we hypothesized that such abundance of not only of parasite components—but also necrosis-derived molecules—would impact directly on liver expression of different inflammatory genes^63–65^. In fact, as depicted in Fig. 1F, several immune-system related pathways were upregulated during *P. chabaudi* infection, as compared to controls. Using a polled analysis strategy where general pathways were grouped together, we detected enhanced expression key pro-inflammatory gene groups, including Toll-like receptors, inflammasomes, scavenger receptors and others. Also, genes involved in phagocytosis (scavenger receptors and complement) were also upregulated, which was accompanied by a sustained elevation of hematopoiesis and cell cycle genes, including pro-inflammatory cell death modalities such as pyroptosis. Corroborating our clinical data on liver metabolism, genes belonging to liver metabolic function, including cytochrome P450 pathways, were downregulated. In contrast—probably due to red blood cell parasitism by *P. chabaudi*—several genes related to iron metabolism were higher expressed in liver samples, which exception of Bdh2 (cytosolic ketone bodies metabolism) and Hamp2 (regulation of systemic iron metabolism). These data, together, demonstrated that not only liver metabolism, but especially hepatic immune system, are dramatically altered due to *Plasmodium* infection. An expanded view of all genes and pathways analyzed can be appreciated in Fig. 1G.

### Alterations in different hepatic immune cell populations, along with massive Kupffer cell depletion, are a hallmark of *Plasmodium* infection

**Figure 2:**
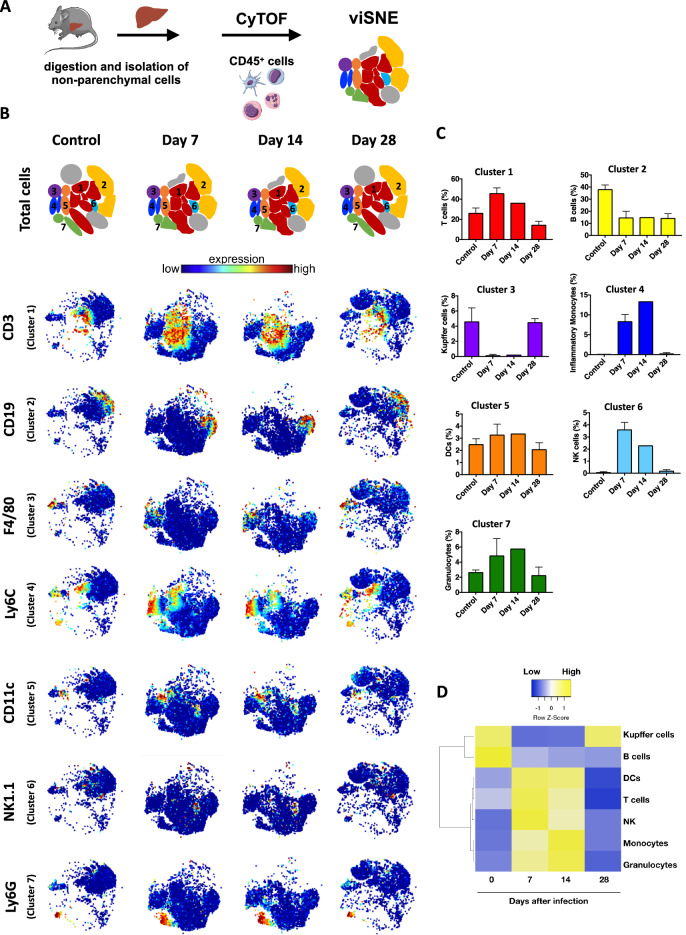
Identification of liver immune cells by CyTOF. (**A**) Schematic representation of liver nonparenchymal cells and clustering after CyTOF Cytobank analysis. The scheme was partly generated using Sevier Medical Art, provided by Sevier, licensed under a Creative Commons Attribution 3.0 unported license. (**B**) Representation of viSNE analysis showing clusters and the expression of several different surface markers that enable the distinction of immune populations. (**C**) Representative bar graph indicating the frequencies of immune cell populations. Data are represented as mean. (**D**) Heatmap illustrating the differential expression of cell population markers in different time points after infection.

Based on higher expression of a plethora of genes related to immune system in the progression of *Plasmodium* infection, we next used a high dimensional immunophenotyping strategy based on Time-of-flight flow cytometry (CyTOF) to map the frequency of different immune cell populations (CD45^+^ events) within the liver. Data analysis of CyTOF results should take into account both dot plots and frequency results. For this, liver non-parenchymal cells were isolated and incubated with a mix of 42 isotope-conjugated antibodies, allowing a precise identification and quantification under single cell mass spectrometry (Fig. 2A). As shown in viSNE analysis (Fig. 2B,C), control mice have a minor population of granulocytes, inflammatory monocytes and NK cells under baseline conditions, along with an expressive population of B cells, T cells, Kupffer cells (KCs) and DCs. Following infection by *P. chabaudi*, a different scenario was observed, since at the peak of parasitemia we found a significant increase of hepatic granulocytes, T cells and NK cells, which was accompanied by reduction in B cells frequency (Fig. 2C,D). Interestingly, KCs (F4/80^hi^ CD11b^+^ Ly6C^low^ DC-SIGN^-^) were the most affected population during infection, since we detected an almost full depletion 7 to14 days day post-infection, which returned to normal values only at 28 days of disease (Fig. 2C,D). In complete coincidence with these data, inflammatory monocytes infiltrated within the liver when KCs were absent, disappearing after 28 days post infection—a timepoint that KC numbers returned to normal. This suggest that *Plasmodium* infection causes an acute immune response in the liver which is not only related to the expression of inflammatory genes, but also with major changes in liver leukocyte populations—including an abrupt impact on hepatic reticuloendothelial system. A summarized view of hepatic leukocyte dynamics can be seen at the heatmap (Fig. 2D).

**Figure 3:**
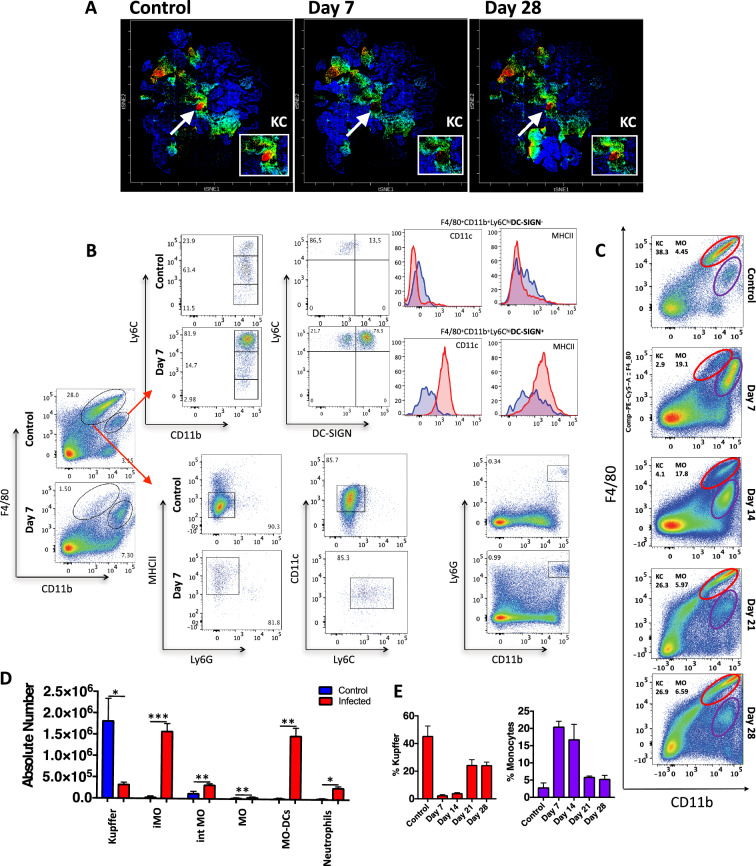
Number of Kupffer cells in the liver during acute phase of *P. chabaudi*. (**A**) Density dot plot CyTOF showing clustered population based on F4/80 expression. (**B**) Flow cytometry gating strategy to immunophenotyped liver non-parenchymal cells. (**C**) Flow cytometry kinetic of Kupffer cells F4/80^high^CD11b^+^ and (**D**) Number of different leukocytes per liver following *P. chabaudi* infection on day 7 post-infection (n = 5). (**E**) Bar graphs represent the percentage of Kupffer cells and monocytes following *P. chabaudi* infection. The data shown are representative of 4 independent experiments. Statistical significance comparing infected and non-infected mice (Student’s t test, *p < 0.05, **p < 0.01, ***p < 0.001).

To confirm the dynamics of KC depletion and hepatic inflammatory monocyte traffic during *Plasmodium* infection, we also mapped their frequency using conventional flow cytometry. Focusing on KC/monocyte clusters identified in CyTOF (Fig. 3A), we corroborated that KCs (F4/80^hi^ CD11b^+^ Ly6C^low^ DC-SIGN^-^) are in fact strongly depleted at the peak of parasitemia (7th-14th days), while inflammatory monocytes (F4/80^+^ CD11b^+^ Ly6C^hi^ CD11c^-^ MHCII^low^ DC-SIGN^-^) and monocyte-derived dendritic cells (F4/80^+^ CD11b^+^ Ly6C^hi^ DC-SIGN^+^ CD11c^+^ MHCII^high^) emigrated to the liver at the same time frame (gating strategy in Fig. 3B, KC/iMO gating in Fig. 3C and supplementary Fig. 1C). Both populations also returned to baseline numbers (Fig. 3D) and frequency (Fig. 3E) at the end of disease. Therefore, we identified KCs as a target of *Plasmodium* infection, and their absence could induce a rapid inflammatory monocyte traffic to the liver.

### *Plasmodium* infection leads to long-lasting alterations on Kupffer cell morphology

**Figure 4:**
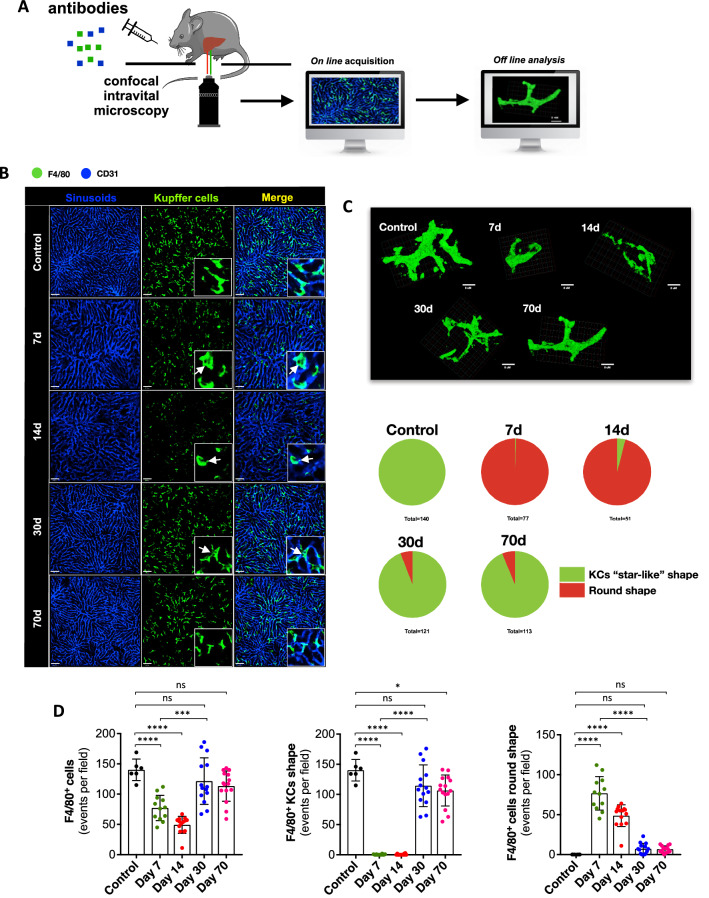
Liver Kupffer cells visualized in vivo during *P. chabaudi* infection. (**A**) Schematic representation of assessment of liver Kupffer cells (KCs) by intravital microscopy (IVM). The scheme was partly generated using Sevier Medical Art, provided by Sevier, licensed under a Creative Commons Attribution 3.0 unported license. (**B**) Liver IVM evidencing the distribution of F4/80^+^ cells (Kupffer cells; anti-F4/80; green) in days 7, 14, 30 and 70 post infection. (**C**) Three-dimensional reconstruction of individual KCs followed by quantification. (**D**) Digital quantification of total F4/80^+^ cells, F4/80^+^ Kupffer “star-like” shape and F4/80^+^ round shape cells. The data shown are representative of 3 independent experiments. Results expressed as mean ± SD. Statistical significance comparing infected and non-infected mice (one-way ANOVA ***p < 0.001, ****p < 0.0001).

Based on our findings using gene expression, CyTOF and flow cytometry, we next imaged KCs/monocytes (F4/80^+^ cells) in their native environment using high resolution confocal intravital microscopy. For this, mice were anesthetized and F4/80^+^ cells and sinusoids were stained in vivo, and both 2D and 3D images were acquired (Fig. 4A). As seen in Fig. 4B, KCs (F4/80^+^ cells with protrusions) are widespread distributed within the liver, inhabiting exclusively the intravascular space, where they can survey blood in real time. For this, KCs have cytoplasmatic arm-like protrusions that can increase their area, allowing a better sampling of molecules and antigens in the circulation, as seen in three-dimensional reconstruction for an intravital microscopy Z-stack (Fig. 4C and Supplementary Video 1). However, upon hepatic invasion of *P. chabaudi*, the frequency of KCs also decreased at the peak of infection (7–14th day), returning to normal values 30 days after (Fig. 4D; chart left). However, regardless the frequency, F4/80^+^ cells morphology was kept altered during malaria (Fig. 4C). In non-infected mice, 100% of F4/80^high^ cells (presumably KCs) exhibit protrusions and are defined as normal when they are imaged as “star-like” cells. In contrast, F4/80^+^ cells from *P. chabaudi* infected mice lost their *bona fide* morphology since they were mostly round cells, lacking the classic protrusions (presumably inflammatory monocytes; Fig. 4C and Supplementary Video 2). Interestingly, despite disease remission (undetected parasites in blood after 30 days post-infection), a residual population of round cells were found within the liver even after 70 days post infection (Fig. 4C,D). This suggest that even if normal cell numbers were reached in the healing phase using CyTOF and flow cytometry approaches, imaging the native environment of immune cells can disclose alterations due to *Plasmodium* infection that are sustained for longer periods, which could also impact on liver ability to clear infections.

### Hemozoin deposition within Kupffer cells triggers cell death by necrosis and ferroptosis

**Figure 5:**
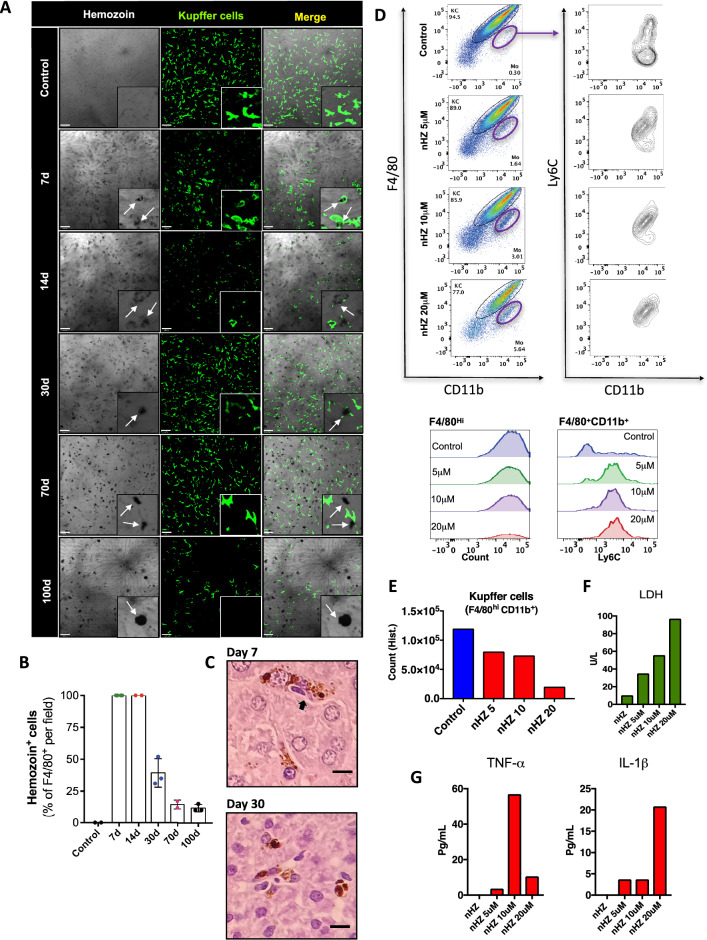
Hemozoin directly promotes cell death of Kupffer cells. (**A**) Intravital microscopy showing the expression and distribution of F4/80^+^ cells (anti-F4/80; green) and hemozoin crystal within the liver. (**B**) Digital quantification of F4/80^+^ cells with hemozoin crystal (n = 2–4). (**C**) Liver histopathology analysis of Kupffer cell carrying hemozoin at day 7 post infection (HE staining, Bar = 16 µm). (**D**) Spleen non-parenchymal cells isolated and cultured overnight with purified hemozoin crystals. (**E**) Absolute number of Kupffer cells, (**F**) LDH release and (**G**) TNF and IL-1β levels after overnight culture of Kupffer cells cultured with hemozoin crystals. The data shown are representative of 3 independent experiments. Data expressed as mean ± SD.

During the intra-erythrocytic stages of malaria infection, up to 80% of the red blood cell cytoplasm can be consumed, and throughout hemoglobin metabolism by *Plasmodium*, significant amounts of hemozoin crystals are formed, phagocytosed and accumulated in KCs as described earlier^39^. It has becoming increasingly clear that stimulation of macrophages with hemozoin can trigger cell activation, including via TLRs, inflammasome ^40–42^, MyD88-independent activation of NF-kappaB and ERK, as well as the release of several subtypes of chemokine^42^. Based on this, we next combined bright field in vivo imaging with fluorescence confocal microscopy to map hemozoin distribution within the liver and merged it with KC location (Fig. 5A). We found that almost 100% of KCs (F4/80^+^ cells) at the peak of infection (7th day) harbor hemozoin inside cytoplasm (assessed by colocalization; Fig. 5A,B), which was sustained up to 14 days after infection. Probably due to the massive KC death during malaria, the percentage of hemozoin-positive cells declined over time (after 30–100 days); however, when we normalized hemozoin positivity to the number of star-shaped cells (presumably mature and phagocytosis-capable cells), it is also reasonable to suggest that the vast majority of F4/80^+^ cells were harboring hemozoin crystals at this timepoint (Fig. 5B). Liver histopathology also confirmed hemozoin uptake by KCs on days 7 and 30 post-infection (Fig. 5C). To further investigate the putative direct cytotoxic effect of hemozoin in KCs, we sorted F4/80^hi^CD11b^+^ cells (KCs; Fig. 5D) and incubated with different concentrations of purified hemozoin (Fig. 5E) and read LDH release in culture medium as an indicative of cell death (Fig. 5F). Interestingly, KCs which were not exposed to hemozoin survived for all experimental protocol; however, exposure to hemozoin induced a dose–response dependent cell death, as assessed by reduced cell counts (Fig. 5E supplementary Fig. 1A) and a significant increase in LDH release (Fig. 5F) and cytokines (Fig. 5G and supplementary Fig. 1B), confirming that hemozoin in vitro (and probably also in vivo) may be a direct cell death agent.

**Figure 6:**
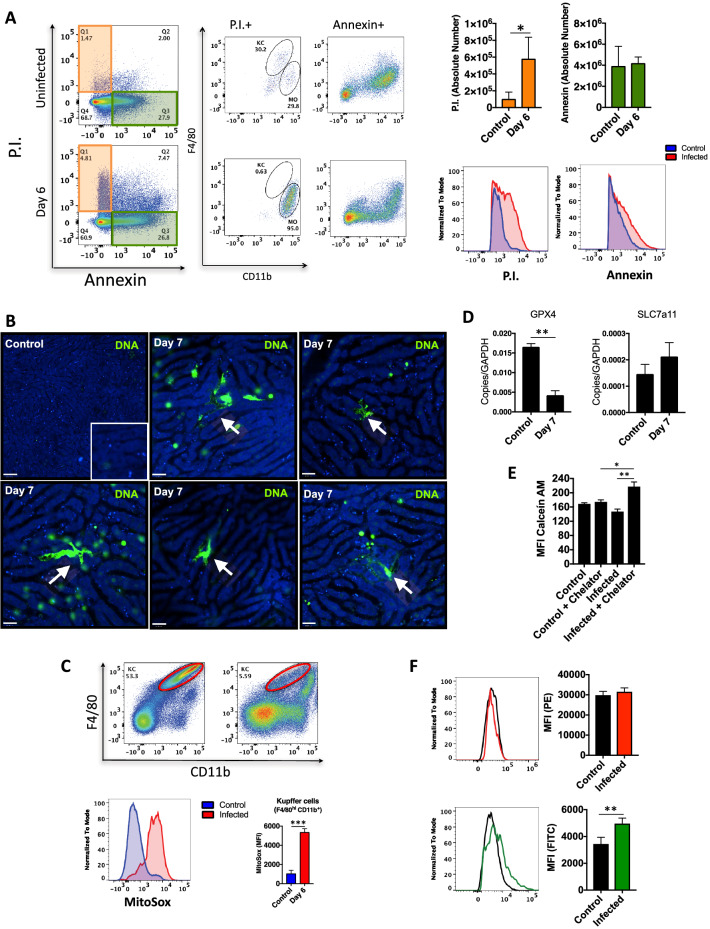
Hemozoin induces Kupffer cells death through ferroptosis. (**A**) Dot plot showing apoptosis and necrosis in the liver. Bars graphs correspond to absolute number of cell death by necrosis or apoptosis (n = 4). (**B**) In vivo extracellular DNA release, (**C**) Mitochondrial superoxide (n = 5), (**D**) GPX4 and SLC7a11 mRNA from liver non-parenchymal cells (n = 6), and (**E**) Intracellular free iron levels in Kupffer cells (n = 3). The data shown are representative of 3 independent experiments. Data expressed as mean ± SD. Statistical significance comparing infected and non-infected mice (one-way ANOVA, * *p* < 0.05, ** *p* < 0.01). (**F**) Lipid peroxidation. Statistical significance comparing infected and non-infected mice (Student’s t test, *p < 0.05, **p < 0.01).

In fact, when we assessed which cell death modality was the most prevalent due to *Plasmodium* infection (Fig. 6A), we found that most KCs were undergoing necrosis (assessed by propidium iodide uptake), but not apoptosis (absence of annexin V staining). To confirm in vivo if KCs were in fact dying due to *Plasmodium* infection and hemozoin deposition, we developed a strategy to map necrosis using confocal intravital microscopy (Fig. 6B). For this, mice received a cell-impermeant DNA-intercalant dye (Sytox green) which strongly stains for dead cells. As seen in Fig. 6B, non-infected mice had almost no DNA staining, even when we image a larger field of view. However, several KCs internalized Sytox green at the peak of infection. The morphology exhibited like amoeboid shape with a largest surface area is specific of KCs, confirming that they are a relevant target for *Plasmodium* infection in vivo. Also, using flow cytometry, we detected that infected KCs had also enhanced production of mitochondrial ROS as compared to controls (Fig. 6C).

Taking into account the presence of iron within hemozoin crystals, we next investigated if KCs could also being depleted due to ferroptosis. Interestingly, gene expression analysis showed a significant downregulation of glutathione peroxidase 4 (*Gpx4*) with concomitant increase of cystine/glutamate antiporter xCT (*Slc7a11*) in infected liver non-parenchymal cells as compared to controls (Fig. 6D). In addition, using a calcein-AM/chelator system (Fig. 6E), we confirmed by flow cytometry that infected KCs harbors significant higher amounts of iron within cytoplasm. In fact, such iron accumulation within KCs was also coincident with enhanced lipid peroxidation, as measured by flow cytometry (Fig. 6F). Together, these findings suggest that ferroptosis might be one of the cell death mechanisms which could explain such overt KC depletion during *Plasmodium* infection.

### Kupffer cell death due to *Plasmodium* infection is a hallmark in enhanced susceptibility to secondary infections

**Figure 7:**
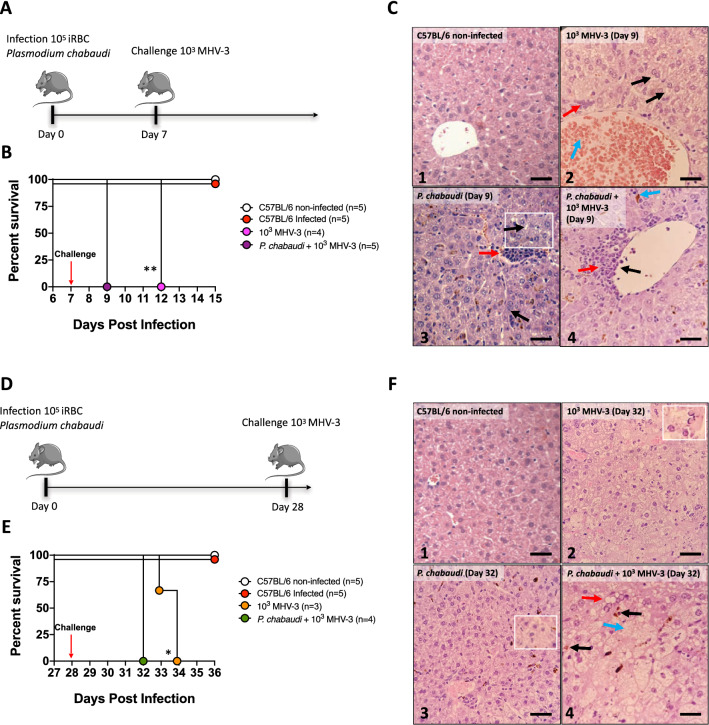
Lethality in *P. chabaudi* mice challenged with MHV-3. (**A**, **D**) Experimental protocol of MHV-3 challenge (Log-rank test, *p < 0.05, **p < 0.01). The scheme was partly generated using Sevier Medical Art, provided by Sevier, licensed under a Creative Commons Attribution 3.0 unported license. Percent survival of mice challenged intraperitoneally with MHV-3 (**B**) 7 and (**E**) 28 days after infection. (**C**) Histology liver sections showing: (C2) Hydropic degeneration (black arrow), hyperemia vessel (blue arrow) and inflammatory cells infiltration (yellow arrow). (C3) Inflammatory focus (yellow arrow), steatosis (black arrow). (C4) Perivascular inflammatory focus (yellow arrow), leukocytes bordering vessel (black arrow) and hemozoin crystals (blue arrow) (HE staining, Bar = 32 µm). (**F**) Histology liver sections showing: (F2) condensed nucleus suggesting apoptosis, (F3) hepatocytes ballooning (HE staining, Bar = 32 µm), (F4) Hemozoin crystals (black arrow), steatosis (yellow arrow), and hydropic degeneration (blue arrow) (HE staining, Bar = 16 µm). Magnification 10X. The data shown are representative of 3 independent experiments.

KCs location within the hepatic sinusoids enables an intimate survey of circulating blood, and this facilitates their major role in clearance of pathogens and microbial products, avoiding the spreading of infections to other organs^24^. Based on our data that KCs are abruptly depleted due to hemozoin internalization, we hypothesized that such deficiency on a key player on liver immunity would impact on host ability to fight secondary infections. Then, we next selected two different models of murine infections that resemble contemporary relevant diseases in public health: coronavirus and *E. coli* infections. For this, mice were first infected with *P. chabaudi* and then challenged with a murine strain of coronavirus (MHV-3, Fig. 7A), and since this mouse hepatitis virus share a common genus with Sars-Cov-2, lessons learnt from MHV-3 could offer mechanistic insights into COVID-19 pathology^43^. When challenged mice only with *Plasmodium*, 100% of mice survived for the whole experimental period (Fig. 7B); however, single intraperitoneally infection with MHV-3 led to full mortality in 5 days post challenge. Interestingly, mice that were challenged with MHV-3 at the peak of *Plasmodium* parasitemia had a more severe variant of liver damage and inflammation, displaying perivascular inflammatory foci (Fig. 7C panel 4; yellow arrow), with accumulation of leukocytes bordering the vessels (Fig. 7C panel 4; black arrow), and hemozoin deposition (Fig. 7C panel 4; blue arrow), succumbing to co-infection significantly earlier (100% lethality at 2 days post infection) (Fig. 7B,C). To understand for how long murine malaria could impair KC function, we also challenged mice when infection was already cleared (28 days post inoculation), knowing that despite undetectable parasitemia, KCs were still harboring intracellular hemozoin at this timepoint (Fig. 7D). In complete agreement, mice that were challenged with MHV-3 at 28 days after *Plasmodium* infection were still more susceptible to disease, presenting significant earlier mortality (Fig. 7E), showing steatosis (Fig. 7F panel 4; yellow arrow), hemozoin crystal (Fig. 7F panel 4; black arrow) and hydropic degeneration (Fig. 7F panel 3; white square, Fig. 7F panel 4; blue arrow) (Supplementary Fig. 2A). Together, these data suggest that failure on KC function induced by *Plasmodium* infection might consist in an aggravating factor in prognosis of relevant respiratory tract diseases, including the current world-wide concerning of Sars-Cov-2 pandemic.

**Figure 8:**
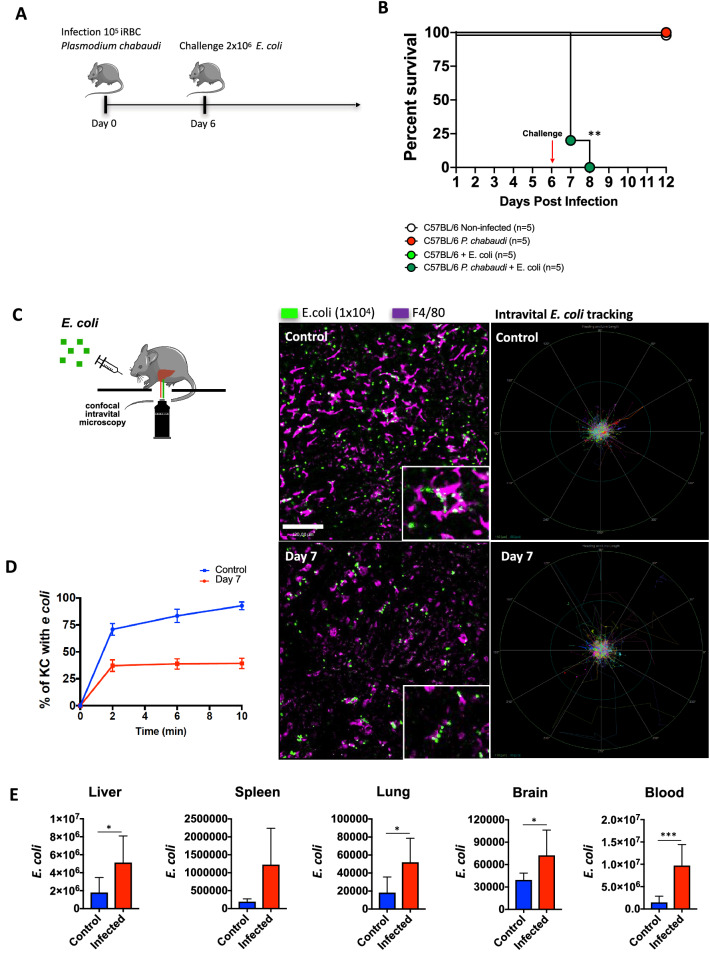
*P. chabaudi* infection leads to reduced ability to fight bacterial infection. (**A**) Experimental protocol of *E. coli* challenge. The scheme was partly generated using Sevier Medical Art, provided by Sevier, licensed under a Creative Commons Attribution 3.0 unported license. (**B**) Percent survival after 6 days of infection followed by *E. coli* challenge. (Log-rank test, **p < 0.01) (**C**) Liver confocal intravital microscopy of KCs (purple; anti-F4/80) and GFP- *E. coli* (green). Digital tracking of *E. coli* displacement within liver sinusoids. (**D**) Digital quantification of Kupffer cells percentage with *E. coli* across the time (n = 4). (**E**) CFU of *E. coli* GPF in liver, spleen, lung, brain and blood (control n = 8, infected n = 4). The data shown are representative of 3 independent experiments. Statistical significance comparing infected and non-infected mice (Student’s t test, *p < 0.05, ***p < 0.001).

We have previously demonstrated that KCs are one of the most significant cells to arrest and clear bacteria in the circulation, and when *E. coli* scape from gut microcirculation and flows within systemic circulation, KCs immediately capture and kill these bacteria in the liver^24^. To further investigate if hemozoin deposition and KC dysfunction could also impair mice ability to face bacterial challenges, we injected mice with *E. coli* at the peak of *plasmodium* parasitemia (Fig. 8A). While mice single infected with *Plasmodium* or *E. coli* completely survived for the whole experimental period, co-infection with bacteria in mice with malaria led to 100% of mortality at 24–48 h after bacterial infection (Fig. 8B). Such enhanced mortality could be—at least in part—explained by a reduced arresting of bacteria within the liver, as demonstrated by off-line digital tracking of *E. coli* behavior in infected mice, as compared to controls (Fig. 8C). As seen in Fig. 8C and Supplementary Video 3, *E. coli* displays a free-flowing behavior within sinusoids in co-infected group, while they were almost instantaneously arrested in mice which are not challenged with *Plasmodium*. Also, the rate of KCs—regardless their reminiscent number—that were able to engulf bacteria was significantly lower in co-infection group as compared to *Plasmodium* only challenged group (Fig. 8D). These data led us to hypothesize that such inefficient KC clearance behavior could predispose systemic bacteria to spread to other organs, fueling widespread infection. In fact, *Plasmodium* infected mice evolved to a massive *E. coli* spread to several organs, including liver, spleen, lung, brain and blood as compared to controls (Fig. 8E). Therefore, KC depletion during *Plasmodium* infection is sufficiently severe to impact in host ability to fight secondary infections. Taken together, our data provided solid mechanistic evidence that hemozoin deposition within liver macrophages, with concomitant inflammation and necrotic cell death, could be a relevant virulence factor during malaria infection, which ultimately predisposes individuals to super infections.

## Discussion

Here we demonstrated that *Plasmodium* infection triggers liver damage and inflammation, which is associated with profound changes in hepatic immunity. In fact, liver has one of the most complex immune cell population within the body, harboring around 25% of all macrophages^44^. In homeostasis, liver is also populated by lymphoid cells—including B, NKT and dendritic cells, and a minor population of neutrophils and patrolling monocytes are found under healthy conditions. However, this scenario is dramatically changed during liver inflammation^28^, and there is a growing body of evidence that alterations on hepatic leukocytes during liver injury are a hallmark of hepatic failure and enhanced susceptibility to infections^24,45^. Therefore, understanding the dynamics of both leukocyte recruitment and cell death during such events might open new venues for treatments and protocols for management of patients with multiple infections.

Malaria is preventable and treatable disease that remains an important cause of morbidity and mortality in African adults, being a major death cause in children under five years. In addition to the human costs of malaria, billions of dollars are spent annually to control and eliminate malaria, representing a significant portion of governmental funding^46^. Importantly, patients diagnosed with malaria can also suffer from other infectious diseases as either super-infections or co-infections, which can enhance mortality rate ^47–49^. We have demonstrated the role of inflammatory monocytes and monocyte derived dendritic cells in the pathogenesis of cerebral malaria and acute respiratory distress syndrome in rodent malaria models^50–52^. Here we demonstrated that *Plasmodium* infection is associated with massive KC depletion, along with sustained alterations on KC morphology and function. Despite KC population being immediately replaced by infiltrated monocytes^28,50^, it has been demonstrated that these monocytic cells—that infiltrate within the liver to occupy niches left by dead KCs—have significant lower phagocytic capacity and express a pro-inflammatory profile^24^.

In fact, when we followed the maturation process of infiltrated monocytes using RNA sequencing and intravital microscopy, we observed that these cells may take up to 30–60 days to display a regular KC behavior, considering both gene expression and bacterial clearance ability. Interestingly, treatment with dexamethasone, which can both reduce hepatic tissue inflammation and increase CSF-1 expression, can accelerate macrophage maturation and rescue KC phagocytic efficacy^24^. Therefore, such maturation window could be—at least in part—responsible for enhanced host susceptibility to secondary infections.

The liver filters blood arisen from mesenteric circulation, receiving also blood from spleen. For this, a significant part of hepatic immune cells is strategically located inside the sinusoids, allowing an intimate contact with pathogens that eventually scape from gut and splenic circulation^18^. However, KCs not only engulf whole parasites—including bacteria and protozoa—but also rapidly arrest particles and molecules within circulation. For instance, injection of latex beads into the bloodstream causes immediate capture and internalization by KCs^53–55^. KCs has also an important role during *Plasmodium* liver stage. The sporozoites selectively recognize and traverse KCs, safely passing through. It suggests that this migration might activate sporozoites for productive invasion of hepatocytes^62^. However, in this work, we have been working in a different stage of *Plasmodium* cycle, the intra-erythrocytic stage. The results presented here show for the first time, using high definition confocal intravital microscopy, that large amounts of hemozoin are stored within KCs, which significantly impacted on cell morphology and function, as assessed by cell depletion, reduced KC protrusions and clearance ability. Such hemozoin accumulation—using in vitro assays—was also associated with imbalanced redox management, as measured by enhanced mitochondrial superoxide production and lipid peroxidation. Therefore, hemozoin accumulation within KCs seems to be more than a simple clearance of *Plasmodium*-derived molecules, but rather a putative virulence mechanism of *Plasmodium* infection.

Hemozoin has been shown to trigger NLRP-3-inflammasome in macrophages, a key event in the pathogenesis of rodent malaria^15,27,40,56^. While our previous study has shown that this event is relevant to neutrophil recruitment to the liver of *P. chabaudi* infected mice^57^, the results presented here show that KCs are still depleted in ASC KO mice. There is an exponential growth in the interest to understand the mechanisms and impacts of cell death due to ferroptosis^58,59,66^. Considering the massive release of heme by bursting infected red blood cells, the iron composition of hemozoin, and intense engulfment of hemozoin by KCs, it is reasonable to hypothesize that ferroptosis, together with other cell death mechanisms, is linked to malaria pathology. Cell death due to ferroptosis is driven by iron-dependent phospholipid peroxidation, which directly impacts on redox homeostasis, intracellular iron concentration, and numerous changes in cell metabolic pathways^60,61^. We observed that *Plasmodium* infected KCs displayed several hallmarks of ferroptosis, which could explain—together with necrotic cell death—the massive disappearance of liver macrophages during malaria. In addition, when we co-infected mice with *E. coli* or murine coronavirus (MHV-3), we found higher mortality rates, which was chronologically associated with massive KC depletion, and also with the spread of pathogens to other organs, including brain, lung and spleen.

Together, our data highlight that accumulation of hemozoin within KC could trigger rapid and massive cell death, with major impacts on liver immunology; therefore, we propose that novel pharmacological strategies that could modulate of ferroptosis and accelerate KC maturation might hold significant potential as an adjuvant treatment to patients with malaria, reducing not only patient morbidity, but also mortality during super-infections.

## Methods

### Ethics statement

The study with malaria patients and healthy donors was approved by the Research Ethics Committee (CEP) from the Research Center of Tropical Medicine (CEP/CEPEM 096/09), the Brazilian National Committee of Research (CONEP/Ministry of Health—15,653), and the Institutional Research Board from the University of Massachusetts Medical School (UMMS) (IRB-ID11116). Informed written consent was obtained from all subjects (*Plasmodium*-infected patients and healthy donors) before enrollment. All methods were carried out in accordance with relevant guidelines and regulations.

### Malaria patients and healthy controls

Patients with acute febrile malaria were treated in the outpatient clinic at the Tropical Medicine Research Center in Porto Velho, Brazil. Up to 100 mL of blood was drawn immediately after confirmation and differentiation of *Plasmodium* infection by a standard peripheral smear and 30–40 days after therapy initiation and confirmed parasitological cure by PCR. Serum from patients infected with *P. vivax* and *P. falciparum* were separated for AST and ALT measurements (age varying from 18 to 60). Non-infected subjects living in Porto Velho were included as controls (age varying from 26 to 59). All methods were carried out in accordance with relevant guidelines and regulations.

### Experimental malaria model

Female C57/BL6 wide-type 8- to 10-week-old mice were obtained from either Animal Facility of the Federal University of Minas Gerais (UFMG), Oswaldo Cruz Foundation (Fiocruz) or University of Massachusetts Medical School. Caspase 1/11^-/-^, TNFαR^-/-^ and IFNγ^-/-^ were obtained from Fiocruz. Wild type mice were bred, reared, and maintained for experiments in micro-isolators in a maximum number of four mice per cage, receiving sterile water and autoclaved chow at animal house from either Fiocruz or University of Massachusetts Medical School. All mice received chow ad libitum and were kept under regular light schedule. Mice were housed under specific pathogen-free conditions. All experiments involving animals were performed in accordance with the American Association for Laboratory Animal Science (AALAS) and Animal Research: Reporting of In Vivo Experiments (ARRIVE) guidelines. All protocols were approved by the Institutional Animal Care and Use Committee (IACUC) at UMMS (ID 2371-15-5) and the Council of Animal Experimentation from Fiocruz and Universidade Federal de Minas Gerais (CEUA protocol 12/21).

The *Plasmodium chabaudi* AS strain (Pc) was used for experimental infections. This strain was kept in our laboratory. Briefly, *Pc* was maintained in C57BL/6 mice by serial passages once a week up to ten times. For experimental infection, blood parasites were counted in a blood smear stained with Giemsa and mice were injected intraperitoneally (i.p.) with 10^5^ infected RBCs. Although animals exhibit signs of disease, lethal infection is not common. Mice were anesthetized i.p. using 100 uL of xylazine (10 mg/Kg) and ketamine (100 mg/Kg).

### Mouse RNA-Seq

RNA-seq was performed in biological replicates (3 mice per group). Liver samples were collected from C57BL/6 mice at day 8 post-infection with *Pc* or non-infected controls. RNA-seq libraries were prepared using the TruSeq Stranded mRNA Kit (Illumina) following the manufacturer’s instructions^32^. Briefly, poly-A containing mRNA molecules were purified using poly-T oligo attached magnetic beads and fragmented using divalent cations. The RNA fragments were transcribed into cDNA using SuperScript II Reverse Transcriptase (Invitrogen), followed by second strand cDNA synthesis using DNA Polymerase I and RNase H. Finally, cDNA fragments then have the addition of a single ’A’ base and subsequent ligation of the adapter. The products were then purified and enriched by PCR using paired-end primers (Illumina) for 15 cycles to create the final cDNA library. The library quality was verified by fragmentation analysis (Agilent Technologies 2100 Bioanalyzer) and submitted for sequencing on the Illumina NextSeq 500 (Bauer Core Facility Harvard University).

### Liver function test

Indocyanine cardiogreen (ICG; Sigma-Aldrich) was measured in mice after 7-, 14- and 28-days post infection. Briefly, mice received 20 mg/kg intravenously (i.v.) of ICG dye, and after 20 min blood was collected. Plasma was diluted and plated in 96-wells polystyrene plate (Nunc, Denmark) and the absorbance measured at 800 nm using a microplate reader (Versa Max)^33^.

### Liver transaminases activity

Liver alanine aminotransferase (ALT) and aspartate transaminase (AST) activity was accessed using a kinetic test (Bioclin, Belo Horizonte, Brazil). Plasma was collected on days 7, 14, 21 and 28 post infection. Samples were processed according to manufacturer’s protocol.

### Liver non-parenchymal cells

Livers were first digested in a collagenase solution (5 mg/liver in 10 mL of RPMI 2% SFB) at 37 °C for 35 min. Then, the solution was added PBS/BSA 0,5% + EDTA in order to inactivate collagenase and cells were differentially separated by centrifugation: 300 × *g* (10 min, 4 °C) disposing supernatants, 60 × *g* (3 min, 4 °C) twice preserving supernatants and finally, 300 × *g* (10 min, 4 °C) disposing supernatants. The samples were filtered thought cells strainer and centrifuged for 300 × *g* (10 min, 4 °C). Red blood cells were lysed using ACK (ammonium chloride potassium) buffer.

### CyTOF

For cytometry by time of flight (CyTOF) experiments, livers were digested in a collagenase solution and cells were differentially separated by centrifugation. 1 × 10^6^ liver non-parenchymal cells were stained with isotope-conjugated antibodies. Events were first gated for CD45 + cells and then CD3^+^, CD19^+^, F4/80^+^, Ly6C^+^, CD11c^+^, NK1.1^+^or Ly6G^+^. Platelets, red blood cells, and hepatic stellate cells were excluded from analysis. Samples were acquired with a Helios Mass Cytometer (Fluidigm, South San Francisco, CA) and data were plotted using viSNE^33^ and Cytobank analysis.

### Gene expression by real-time PCR

Total RNA from liver non-parenchymal cells was extracted by using Trizol Reagent (Invitrogen). After total RNA isolation, the samples were converted in complementary DNA using High-Capacity cDNA Reverse Transcription Kit (Applied Biosystems) according to manufacturer’s instruction. Quantitative PCR was performed with SYBR Green Master Mix (Applied Biosystems). Reactions were carried out in an ABI7500 Real Time PCR System (Applied Biosystems) under standard conditions. Primer sequences: *Gapdh*: F 5’ GGCAAATTCAACGGCACAGT 3’, R 5’ AGATGGTGATGGGCTTCCC 3’; *Gpx4*: F 5’ GCAACCAGTTTGGGAGGCAGGAG 3’, R 5’ CCTCCATGGGACCATAGCGCTTC 3’; *Slc7a11*: F 5’ CCTCTGCCAGCTGTTATTGTT 3’, R 5’ CCTGGCAAAACTGAGGAAAT 3’; *Tnf*: F 5’ CCCTCACACTCAGATCATCTTCT 3’, R 5’ GCTACGACGTGGGCTACAG 3’; *Il1b*: F 5’ ACCTGTCCTGTGTAATGAAAGACG 3’, R 5’ TGGGTATTGCTTGGGATCCA 3’; *Il10*: F 5’ TTTGAATTCCCTGGGTGAGAA 3’, R 5’ GGAGAAATCGATGACAGCGC 3’.

### Intravital confocal microscopy

For in vivo imaging, before surgery mice received a mixture of the following antibodies or fluorescent probes: anti-F4/80 phycoerythrin (0.1 μg/g, clone BM8, BioLegend), anti-CD31 BV-421 (0.15 μg/g, clone 390, BD) or Sytox Green (1 μL, 50 μM, Invitrogen). Infected mice (7 days post-infection) were anaesthetized intraperitoneally (100 mL for each animal) with a mixture of ketamine (37.5 mg/mL, final concentration) and xylazine (2.5 mg/mL, final concentration). Mouse was immobilized using the stereotaxis apparatus. Surgeries were made with minor modifications. Livers were exposed and observed using a confocal microscope (Nikon Eclipse Ti, Objective Plan Apo 20x) outfitted with an A1R confocal head with four different lasers (excitation at four wavelengths: 405, 488, 546, and 647 nm) and emission bandpass filters at 450/50, 515/30, 584/50, and 663/738 nm. Digital quantification was made by Volocity (6.3) (Perkin Elmer, Waltham, MA, USA) and NIS-Elements (Nikon Instruments Inc., Melville, NY, USA)^33–37^ .

### Systemic *Escherichia coli* injection

*Escherichia coli* green fluorescent protein (GFP; ATCC 25922GFP) were cultivated in Luria Bertani medium (MP Biomedicals, Santa Ana, CA). To *in vivo* imaging of *E. coli* capture, mice received 1 × 10^4^ *E. coli* intravenously and were imaged under confocal microscopy for 10 min. For survival experiments, mice were inoculated intravenously with 2 × 10^6^ *E. coli* GFP^24^ .

### Histopathology

Samples of liver were collected, fixed in 10% buffered formalin, dehydrated, cleared, embedded in paraffin, sectioned (3 μm thick), and stained with hematoxylin and eosin and periodic acid-Schiff (PAS) for histopathological studies. Each segment was macroscopically sliced into three fragments. Therefore, each histological slide contained three longitudinal sections. Blind histological analysis of slides was carried out by a minimum of two pathologists.

### Flow cytometry

Livers were removed, non-parenchymal cells were purified (as described above) and counted by using a haemocytometer. Cells were stained with antibodies specific for mouse CD11b (PE-Cy7, clone: M1/70, e-Bioscience), F4/80 (PE-Cy5, clone: BM8, e-Bioscience), CD11c (Alexa 700, clone: N418, e-Bioscience), MHC II (phycoerythrin (PE), clone: AF6–120.1, BD), CD209a (DC-SIGN; APC e-fluor 660, clone: MMD3, e-Bioscience), Ly6c (e-Fluor 450, clone: HK1.4, e-Biosciences), Annexin/PI (BD 556,547) and MitoSox (ThermoFisher M36008) at room temperature for 20 min. Cytometry was performed by using a Fortessa Cytometer (Becton–Dickinson) and analysed with FlowJo software v10.6.1.

### Cell sorting

Liver from non-infected mice were harvested and non-parenchymal cells stained with CD11b (PE-Cy7, clone: M1/70, e-Bioscience) and F4/80 (PE-Cy5, clone: BM8, e-Bioscience) then submitted to purification by using a cell sorting ARIA (BD). These cells were first gated on FSC-H/FSC-A, to avoid doublets and then on SSC-A/FSC-A. Next, gated on F4/80^hi^/CD11b^+^. The gated cells were sorted and collected into fresh new tube. After centrifuge, cells were used for culture with *P. chabaudi* purified natural hemozoin.

### Purified *P. chabaudi* natural hemozoin (nHZ)

Eight IFNγ^-/-^ mice infected spleens were processed to obtain natural *P. chabaudi* hemozoin. Spleens were lysed with ACK buffer for 10 min. After two washes (1800 rpm, 5 min, 4 °C), cells were resuspended in 20 mL RPMI and sonicated for 10 min. Then, they were submitted 5 rounds of freeze (liquid nitrogen) and thaw (37 °C), washed and filtered thought cells strainer. The samples were washed one more time and resuspended in 500 μL. Hemozoin was quantified using Heme Assay Kit, cat number MAK316 (Sigma Aldrich, St. Louis, MO). Liver non-parenchymal cells were stimulated (5 × 10^5^) with nHZ (5 μM, 10 μM or 20 μM) overnight in polypropylene round-bottom non adherent tube.

### Cytokine and LDH measurements

Measurements in supernatant of Kupffer cells cultures with nHZ were performed using commercially available ELISA Duoset kit for IL-1β, TNF (ThermoFisher) and LDH (Bioclin). Absorbance was read with SOFTmaxPRO V4.3.1 LS.

### Intracellular iron quantification

Labile intracellular iron was measured by using the calcein acetoxymethyl (AM) ester quenching method (Invitrogen). Briefly, liver non-parenchymal cells were purified and 1 × 10^6^ cells were stained with antibodies specific for mouse CD11b (PE-Cy7, clone: M1/70, e-Bioscience) and F4/80 (PE-Cy5, clone: BM8, e-Bioscience) for 20 min at room temperature. Then, cells were washed 2 times with PBS FACS and incubated with calcein AM (100 nM) and/or an iron chelator deferoxamine (250 μM) (Sigma-Aldrich) at 37 °C for 30 min. Calcein AM fluorescence was analyzed immediately by flow cytometry, using a Fortessa Cytometer (Becton–Dickinson) and Flowjo software v10.6.1.

### Lipid peroxidation assay

Lipid peroxidation in Kupffer cells was assessed by using lipid peroxidation assay kit (Abcam Ab243377). 1 × 10^6^ of liver non-parenchymal cells were stained with antibodies specific for mouse CD11b (PE-Cy7, clone: M1/70, e-Bioscience) and F4/80 (PE-Cy5, clone: BM8, e-Bioscience) for 20 min at room temperature. Then, cells were washed 2 times with PBS FACS and incubated with 1 × lipid peroxidation sensor for 30 min at 37 °C. The cells were washed 3 times with HHBS and analysed using a Fortessa Cytometer (Becton–Dickinson) and FlowJo software v10.6.1.

### Bacterial load (Colony forming units)

For colony-forming unit (CFU) estimation, mice intravenously received 2 × 10^6^ GFP- *E. coli* per mouse. 18 h after injection, blood, liver, spleen, brain and lung were harvest and samples were filtered thought cells strainer and centrifuged for 1800 × *g* (5 min, 4 °C). Red blood cells were lysed using ACK (ammonium chloride potassium) buffer. After three rounds of washes, the samples were incubated with counting beads (CountBright absolute counting beads; Invitrogen) following the manufacturer’s recommendations and fluorescence was immediately analyzed by flow cytometry, using a Fortessa Cytometer (Becton–Dickinson) and FlowJo software v10.6.1.

### Statistical analysis

All data were analyzed using Graphpad Prism 7.0 Software. Comparisons were performed using a one-way analysis of variance (ANOVA) or Student’s t test for data analysis and generation of *P*-values. Mann–Whitney or Kruskal–Wallis testing was used for non-parametric analysis when data did not fit a Gaussian distribution. Analysis of variance via post-Tukey’s test for multiple comparisons was used for comparing more than two samples. A *p* < 0.05 value was considered statistically significant. All data are represented as median with individual data points representing individual samples. Bar graph data show SD error bars.

## Supplementary Information

Supplementary Video 1.

Supplementary Video 2.

Supplementary Video 3.

Supplementary Information 1.

## Data Availability

RNA Seq Data GEO (https://www.ncbi.nlm.nih.gov/geo/query/acc.cgi?acc=GSE109908) accession number is GEO: GSE109908.
