# Supplementary material for: Uptake of Plasmodium chabaudi hemozoin drives Kupffer cell death and fuels superinfections
Source: Sci Rep. 2022 Nov 17;12:19805. doi: 10.1038/s41598-022-23858-7 (PMC9671901; doi:10.1038/s41598-022-23858-7)
Supplement: Supplementary file 4 — Supplementary Information 1. [file 41598_2022_23858_MOESM4_ESM.pdf]

Uptake of *Plasmodium* hemozoin drives Kupffer cell death and fuels superinfections

Isabella C. Hirako[1,2], Maísa Mota Antunes[4], Rafael Machado Rezende[3], Natália Satchiko Hojo-Souza[1], Maria Marta Figueiredo[6], Thomaz Dias[7], Helder Nakaya[7], Gustavo Batista Menezes[4*] and Ricardo Tostes Gazzinelli[1,2,5,8*]

Instituto René Rachou, Fundação Oswaldo Cruz, Belo Horizonte, MG, Brazil[1]; Division of Infectious Diseases and Immunology, University of Massachusetts Medical School, Worcester, MA, USA[2]; Ann Romney Center for Neurologic Diseases, Brigham and Women's Hospital, Harvard Medical School, Boston, MA, USA[3]; Center for Gastrointestinal Biology, Departamento de Morfologia, Instituto de Ciências Biológicas, Universidade Federal de Minas Gerais, Belo Horizonte, MG, Brazil[4]; Departamento de Bioquímica e Imunologia, Instituto de Ciências Biológicas, Universidade Federal de Minas Gerais, Belo Horizonte, MG, Brazil[5]; Universidade Estadual de Minas Gerais, Divinópolis, MG, Brazil[6]; Escola de Ciências Farmacêuticas – Universidade de São Paulo, São Paulo, SP, Brazil[7]; Plataforma de Medicina Translacional, Fundação Oswaldo Cruz / Faculdade de Medicina de Ribeirão Preto, Ribeirão Preto, SP, Brazil[8].

*These authors jointly supervised this work.

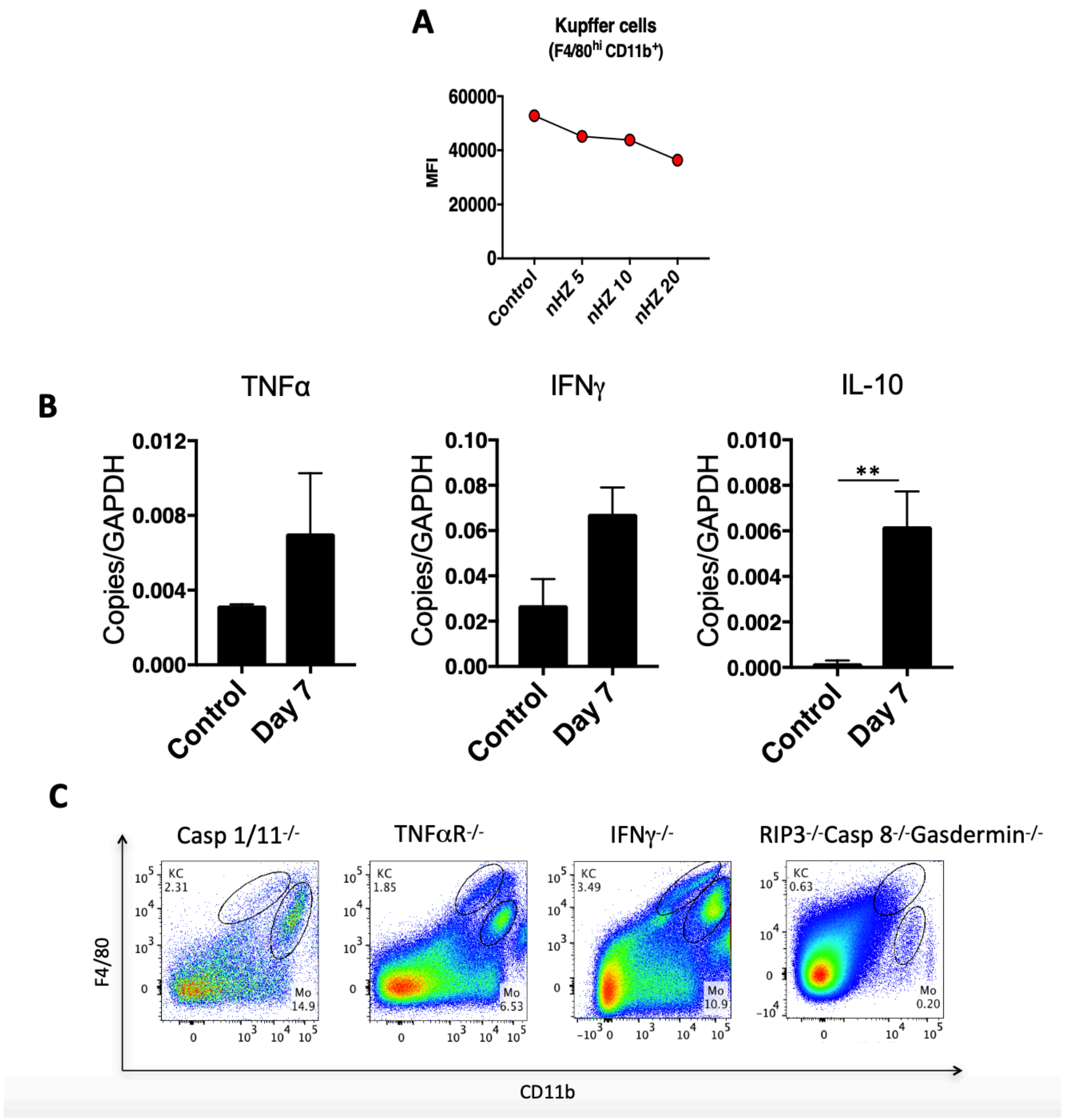

Supplementary Fig. 1: Kupffer cell death and cytokine deficient mice. (A) Line graphs correspond to MFI of F4/80^{high} Kupffer cells after overnight culture with natural hemozoin crystals. (B) TNF-α, IFN-γ and IL-10 mRNA expression in isolated liver nonparenchymal cells. (C) Percentage of Kupffer cells from Caspase 1/11$^{-/-}$, TNFαR$^{-/-}$, IFNγ$^{-/-}$ and RIP3$^{-/-}$ Casp 8$^{-/-}$ Gasdermin$^{-/-}$ mice 7 days post *P. chabaudi* infection. Data are represented as mean ± SD. Statistical significance comparing infected and non-infected mice. (Student's t test, $^{**}p < 0.01$).

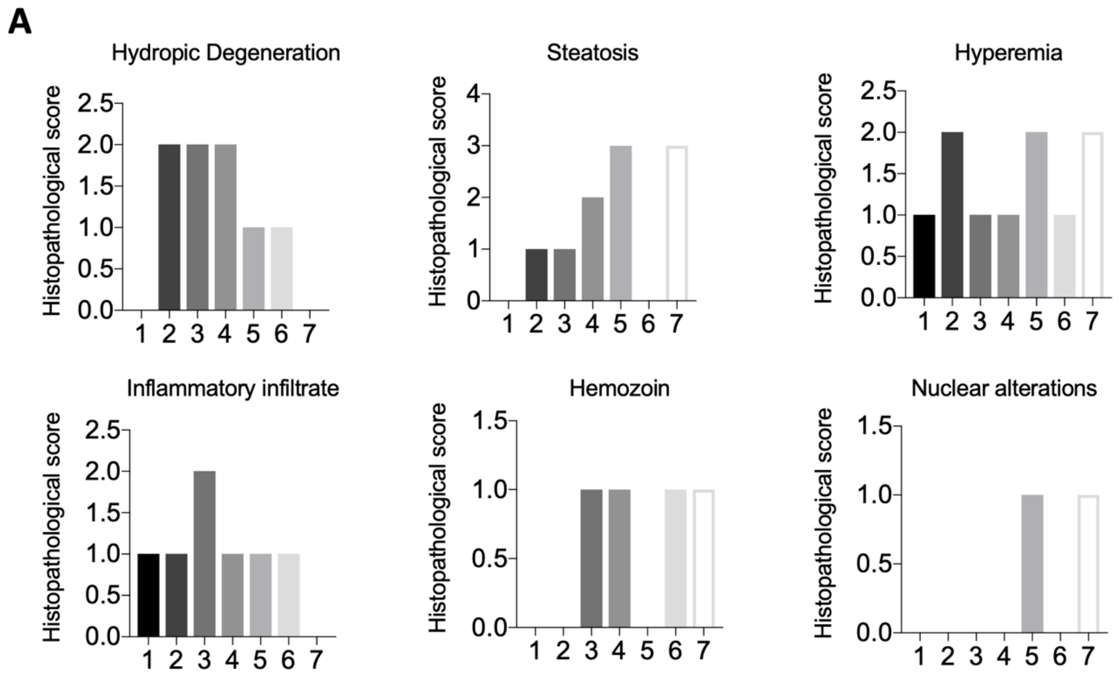

Supplementary Fig. 2: Histopathological scores. (A1) Hydropic degeneration, (A2) Steatosis, (A3) Hyperemia, (A4) Inflammatory infiltrate, (A5) Hemozoin and (A6) Nuclear alterations. Samples: 1) C57BL/6 non-infected. 2) 10^3 MHV-3. 3) *P. chabaudi* (day 9). 4) *P. chabaudi* (day 9) + 10^3 MHV-3. 5) 10^3 MHV-3. 6) *P. chabaudi* (day 32). 7) *P. chabaudi* (day 32) + 10^3 MHV-3.

Supplementary Video 1: Kupffer cells localization in sinusoids and cytoplasmatic arm-like protrusions. Three-dimensional reconstruction for an intravital microscopy Z-stack showing Kupffer cells (anti-F4/80; green) localization in sinusoids (anti-CD31; blue) and cytoplasmatic arm-like protrusions that can increase their area, allowing a better sampling of molecules and antigens in the circulation. 20x Objective.

Supplementary Video 2: Changes on morphology of Kupffer cells over *P. chabaudi* infection. Three-dimensional reconstruction for an intravital

microscopy Z-stack showing transition of morphology of an Kupffer "star-like" shape cell (anti-F4/80; green) to a round-shape morphology, lacking the classic protrusions. 20x Objective.

Supplementary Video 3: Catching of *E. coli* by Kupffer cells of uninfected and infected mice. Real-time movie showing *E. coli* (e-GFP; green) free flowing within sinusoids in co-infected group, while they were almost instantaneously arrested by Kupffer cells (anti-F4/80; purple) in mice which are not challenged with *Plasmodium*. 20x Objective.
